# Identifying delays in healthcare seeking and provision: The Three Delays-in-Healthcare and mortality among infants and children aged 1–59 months

**DOI:** 10.1371/journal.pgph.0002494

**Published:** 2024-02-08

**Authors:** Elisa Garcia Gomez, Kitiezo Aggrey Igunza, Zachary J. Madewell, Victor Akelo, Dickens Onyango, Shams El Arifeen, Emily S. Gurley, Mohammad Zahid Hossain, Md Atique Iqbal Chowdhury, Kazi Munisul Islam, Nega Assefa, J. Anthony G. Scott, Lola Madrid, Yenenesh Tilahun, Stian Orlien, Karen L. Kotloff, Milagritos D. Tapia, Adama Mamby Keita, Ashka Mehta, Amilcar Magaço, David Torres-Fernandez, Ariel Nhacolo, Quique Bassat, Inácio Mandomando, Ikechukwu Ogbuanu, Carrie Jo Cain, Ronita Luke, Sorie I. B. Kamara, Hailemariam Legesse, Shabir Madhi, Ziyaad Dangor, Sana Mahtab, Amy Wise, Yasmin Adam, Cynthia G. Whitney, Portia C. Mutevedzi, Dianna M. Blau, Robert F. Breiman, Beth A. Tippett Barr, Chris A. Rees, Child Health and Mortality Prevention Surveillance Network

**Affiliations:** 1 Emory University School of Medicine, Emory University, Atlanta, Georgia, United States of America; 2 Kenya Medical Research Institute-Center for Global Health Research, Kisumu, Kenya; 3 Global Health Center, Centers for Disease Control and Prevention, Atlanta, Georgia, United States of America; 4 Centers for Disease Control and Prevention-Kenya, Kisumu, Kenya; 5 Kisumu County Department of Health, Kisumu, Kenya; 6 International Centre for Diarrhoeal Disease Research Bangladesh (icddr,b), Dhaka, Bangladesh; 7 Bloomberg School of Public Health, Johns Hopkins University, Baltimore, Maryland, United States of America; 8 London School of Hygiene and Tropical Medicine, London, United Kingdom; 9 College of Health and Medical Sciences, Haramaya University, Harar, Ethiopia; 10 Hararghe Health Research, Haramaya University, Harar, Ethiopia; 11 College of Medicine and Health Sciences, University of Hargeisa, Hargeisa, Somaliland; 12 Department of Paediatrics, Vestfold Hospital Trust, Tønsberg, Norway; 13 Department of Pediatrics, Center for Vaccine Development and Global Health, University of Maryland School of Medicine, Baltimore, Maryland, United States of America; 14 Centre pour le Développement des Vaccins-Mali, Bamako, Mali; 15 Centro de Investigação em Saúde de Manhiça (CISM), Maputo, Mozambique; 16 ISGlobal – Hospital Clínic, Universitat de Barcelona, Barcelona, Spain; 17 Institució Catalana de Recerca I Estudis Avançats (ICREA), Barcelona, Spain; 18 Pediatrics Department, Hospital Sant Joan de Déu, Universitat de Barcelona, Esplugues, Barcelona, Spain; 19 Consorcio de Investigación Biomédica en Red de Epidemiología y Salud Pública (CIBERESP), Madrid, Spain; 20 Instituto Nacional de Saúde, Ministério de Saúde, Maputo, Moçambique; 21 Crown Agents, Freetown, Sierra Leone; 22 World Hope International, Freetown, Sierra Leone; 23 Ministry of Health and Sanitation, Freetown, Sierra Leone; 24 UNICEF, Freetown, Sierra Leone; 25 South African Medical Research Council Vaccines and Infectious Diseases Analytics Research Unit, University of the Witwatersrand, Johannesburg, South Africa; 26 Department of Obstetrics and Gynaecology, Rahima Mossa Mother and Child Hospital, University of the Witwatersrand, Johannesburg, South Africa; 27 Department of Obstetrics and Gynaecology, Chris Hani Baragwanath Academic Hospital, University of the Witwatersrand, Johannesburg, South Africa; 28 Global Health Institute, Emory University, Atlanta, Georgia, United States of America; 29 Hubert Department of Global Health, Rollins School of Public Health, Emory University, Atlanta, Georgia, United States of America; 30 Nyanja Health Research Institute, Salima, Malawi; 31 Department of Pediatrics, Emory University School of Medicine, Atlanta, Georgia, United States of America; 32 Children’s Healthcare of Atlanta, Atlanta, Georgia, United States of America; PLOS: Public Library of Science, UNITED STATES

## Abstract

Delays in illness recognition, healthcare seeking, and in the provision of appropriate clinical care are common in resource-limited settings. Our objective was to determine the frequency of delays in the “Three Delays-in-Healthcare”, and factors associated with delays, among deceased infants and children in seven countries with high childhood mortality. We conducted a retrospective, descriptive study using data from verbal autopsies and medical records for infants and children aged 1–59 months who died between December 2016 and February 2022 in six sites in sub-Saharan Africa and one in South Asia (Bangladesh) and were enrolled in Child Health and Mortality Prevention Surveillance (CHAMPS). Delays in 1) illness recognition in the home/decision to seek care, 2) transportation to healthcare facilities, and 3) the receipt of clinical care in healthcare facilities were categorized according to the “Three Delays-in-Healthcare”. Comparisons in factors associated with delays were made using Chi-square testing. Information was available for 1,326 deaths among infants and under 5 children. The majority had at least one identified delay (n = 854, 64%). Waiting >72 hours after illness recognition to seek health care (n = 422, 32%) was the most common delay. Challenges in obtaining transportation occurred infrequently when seeking care (n = 51, 4%). In healthcare facilities, prescribed medications were sometimes unavailable (n = 102, 8%). Deceased children aged 12–59 months experienced more delay than infants aged 1–11 months (68% vs. 61%, *P* = 0.018). Delays in seeking clinical care were common among deceased infants and children. Additional study to assess the frequency of delays in seeking clinical care and its provision among children who survive is warranted.

## Introduction

In 2021, more than 5 million children died before their fifth birthday worldwide and over 80% of these deaths occurred in sub-Saharan Africa and Southeast Asia [1]. Given the burden of childhood mortality in these regions, emphasis has been placed on improving demand and access to high quality clinical care. However, delayed healthcare seeking, long distances to healthcare facilities, and limited laboratory and radiographic capabilities continue to be common in these regions and contribute to shortcomings in high-quality clinical care [2–4]. We have previously described that many deaths among infants in children in the Child Health and Mortality Prevention Surveillance (CHAMPS) network may have been averted through improved health-seeking behaviors [5]. However, a detailed description of the specific aspects of needed changes to health-seeking behavior to prevent childhood mortality is lacking.

The “Three Delays-in-Healthcare” model was developed/proposed to characterize the delays leading to maternal mortality, with respect to the time for: 1) the decision to seek medical care, 2) traveling to and reaching a healthcare facility, and 3) receiving adequate clinical care once in a healthcare facility [6]. Though previously utilized to understand major delays leading to maternal mortality [6], surgical care [7, 8], and adult mortality in resource-limited settings [9, 10], this model has been used in few instances to understand delays in healthcare seeking and provision for children who urgently require medical treatment [11–13].

An understanding of healthcare seeking behaviors among caregivers of deceased infants and children is a necessary step toward developing feasible interventions to reduce childhood deaths in high mortality regions and achieve the Sustainable Development Goal of ending preventable childhood deaths by the year 2030 [14]. Our objective was to determine the frequency of delays in the “Three Delays-in-Healthcare”, and factors associated with delays, among deceased infants and children in seven countries with high childhood mortality.

## Materials and methods

### Study design

We conducted a retrospective, descriptive study using data collected on deceased infants and children aged 1–59 months who enrolled in Child Health and Mortality Prevention Surveillance (CHAMPS) between December 2016 and February 2022. We accessed data for the present study on March 12, 2022. We used the “Three Delays-Healthcare” framework to categorize and contextualize delays in healthcare seeking and provision [6]. The ethical review committee of each site approved CHAMPS mortality surveillance and the use of the data used in this study. The use of CHAMPS data has also been approved by the Institutional Review Board at Emory University Rollins School of Public Health.

### Data sources

CHAMPS has been described previously [15, 16]. In brief, CHAMPS is a multi-country network that conducts prospective community and healthcare center childhood mortality surveillance in regions with disproportionately high burden of mortality in children aged <5 years (i.e., Baliakandi and Faridpur, Bangladesh; Harar, Haramaya, and Kersa, Ethiopia; Kisumu and Siaya, Kenya; Bamako, Mali; Manhiça and Quelimane, Mozambique; Makeni, Sierra Leone; Soweto, South Africa). Community health volunteers in each included community conduct community mortality surveillance and healthcare providers and hospital staff conduct hospital surveillance. When deceased children are identified, caregivers are approached for potential enrollment for extensive postmortem examinations of the deceased child including postmortem histopathological and microbiological analyses including immunohistochemistry staining and molecular pathogen identification through extensive polymerase chain reaction (PCR) testing for 126 different pathogens (TaqMan array; ThermoFisher Scientific, Waltham, MA, USA). Stillbirths and deaths in live-born children under 5 years of age in CHAMPS catchment areas were eligible for enrollment in CHAMPS (either cases that underwent minimally invasive tissue sampling and had verbal autopsy and clinical data or cases with verbal autopsy and clinical data alone). However, the present analysis is restricted to information from infants and children. If caregivers consented, CHAMPS staff at each site collected demographic, verbal autopsy, and clinical information for each enrolled death. Here, we included cases of death that occurred both in healthcare facilities and those that occurred outside health facilities in the community.

As part of CHAMPS routine data collection and as soon as possible following the death, local study staff, who did not provide clinical care, comprehensively reviewed all available medical records for clinic visits and hospital admissions prior to the infant’s or child’s death to document the clinical care an infant or child received, if this information was available. These data include information about the frequency of clinic visits and hospital admissions, clinical care provided, and barriers to the provision of clinical care prior to death.

CHAMPS staff completed the 2016 World Health Organization (WHO) Verbal Autopsy form with caregivers of enrolled deceased infants and children within 2–4 weeks of the death [15, 17]. The 2016 WHO Verbal Autopsy is a validated tool for obtaining context for events that preceded death and includes questions with discrete answers (e.g., duration of symptoms, clinical encounters, etc.) as well as a narrative field in which caregivers describe events occurring before the death [17].

Deaths identified within 24 hours of enrollment (or 72 hours if the body was refrigerated) were eligible for specimen collection and testing. For eligible cases, a consenting team (i.e., a staff trained in providing support and counseling to families and a health worker) was dispatched to the family as soon as possible to confirm eligibility and obtain written consent to all CHAMPS procedures [15]. For these deaths, CHAMPS teams collected minimally invasive tissue samples (MITS) from lungs, brain, and liver. Additionally, CHAMPS staff collected swabs of the rectum and upper airway, blood, and cerebrospinal fluid samples. Specimens underwent culture, polymerase chain reaction testing to identify infectious causes of death and tissues were reviewed by pathologists.

In each site, a Determination of Cause of Death (DeCoDe) panel consisting of local physicians, public health professionals, and other healthcare providers performed a standardized process to assign causes of death for each case enrolled in CHAMPS who underwent MITS [18]. The DeCoDe panel analyzed all available data from verbal autopsies, clinical summaries (if available), and histopathologic and microbiologic investigations to determine the causes for each death. CHAMPS uses the WHO International Statistical Classification of Diseases and Related Health Problems, Tenth Revision (ICD-10) and the WHO application of ICD-10 deaths during the perinatal period (ICD-PM) to assign causes of death [19, 20]. Multiple causes of death are common in CHAMPS and causes are categorized as immediate, underlying, and co-morbid causes of death [21]. Once causes of death were determined, CHAMPS staff contacted caregivers to provide information about the causes of death.

### Inclusion and exclusion criteria

Our analysis included all infants and children aged 1–59 months whose caregivers agreed to enroll in CHAMPS during the study period and had verbal autopsy or clinical summary data available. To ensure that CHAMPS includes a representative sample of deaths that occur in the included catchment areas, prospective community and healthcare center childhood mortality surveillance is conducted in which notifications of all deaths among children aged <5 years are targeted. We excluded stillbirths, neonates, and infants who were never discharged from the hospital after birth as healthcare seeking differs in these populations, largely depend on maternal healthcare seeking patterns, and warrant a detailed independent description to inform interventions for pregnant mothers that may differ from those that cater to caregivers of infants and children. We also excluded infants and children who had insufficient information in their verbal autopsy or clinical summaries to describe healthcare seeking or clinical care received.

### Data extraction

We created a list of variables that corresponded to each of the “Three Delays-in-Healthcare” framework as documented in previous studies and in consultation with experienced members of CHAMPS [7, 22, 23]. We then extracted data corresponding to these variables, including information related to: 1) when a caregiver recognized signs or symptoms for infants and children in the home, including time from recognition to deciding to seek care (i.e., Delay 1); 2) potential barriers related to transportation to seek clinical care described by caregivers (i.e., Delay 2); and 3) treatment once a child reached a facility, including the availability of healthcare providers, treatments provided including issues with their availability, and affordability of diagnostics and therapeutics (i.e., Delay 3).

We reviewed narrative fields in verbal autopsies and clinical summaries to extract information related to the three delays. A subset of 10% of all cases were independently reviewed by a second reviewer to ensure reproducibility in data extraction from narrative fields. Questions regarding variable classification were discussed by the first and last authors until consensus was achieved.

### Statistical analysis

We calculated descriptive statistics for demographics and for delays in the “Three Delays-in-Healthcare” framework. We compared frequencies of delays by the deceased’s age (i.e., 1–11 months vs. 12–59 months) because prior studies suggest healthcare seeking and provision delays may be more common in older children [24–26]. Additionally, since delays in healthcare seeking may be more common for female children [2, 22, 25], we compared the frequency of delays by participant sex [24, 27, 28]. We also compared delays by site, given anticipated variability in healthcare seeking behaviors between each country. Lastly, because we postulated that healthcare seeking may vary by the infant’s or child’s diagnosis, we evaluated the odds of experiencing at least one delay between DeCoDe determined causes of death [29, 30]. *P* values for the cause of death comparison were corrected for multiple comparisons using the Benjamin-Hochberg procedure, and false discovery rate–adjusted *P* values (*q*-values) were used for evaluating significance [31]. Statistical significance was defined as *q* ≤0.10 (i.e., controlling the false discovery rate [FDR] at 10%). All statistical analyses were performed using R software, version 4.1.2 (R Foundation for Statistical Computing).

## Results

**Fig 1:**
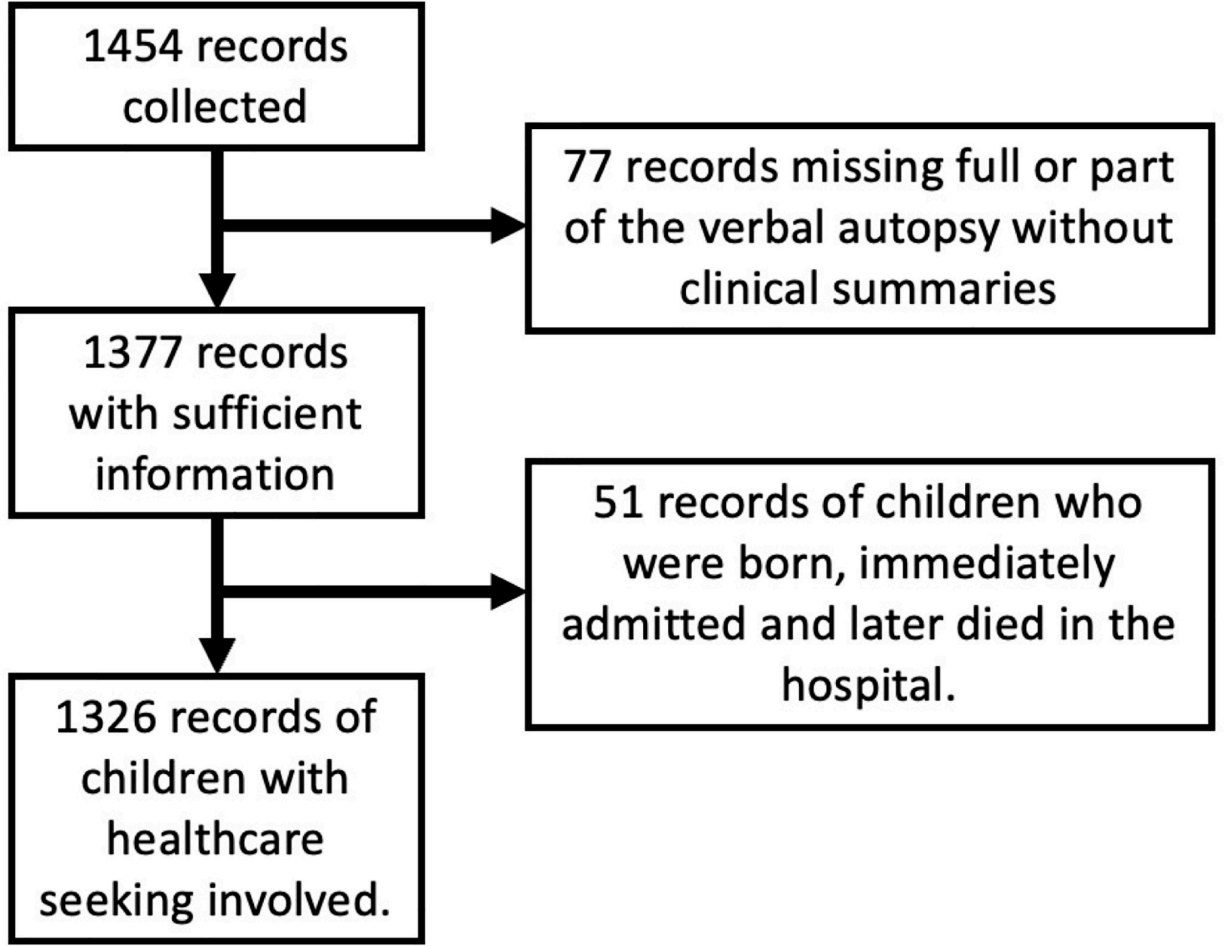
PRISMA flow diagram for case selection.

There were 1,454 infants and children aged 1–59 months who died during the study period. Of those, 77 (5%) had insufficient information in their verbal autopsy or clinical summaries to describe healthcare seeking and provision and 51 (4%) were born and died in the hospital without discharge. The remaining 1,326 (91%) children were included in our analyses (Fig 1). The 77 infants and children who were excluded for insufficient information did not differ from those included in our analyses by age at death (*P* = 0.97) or sex (*P* = 0.37) but were more commonly deaths that occurred in the community outside healthcare facilities than those who were included (n = 54, 71.1% and n = 506, 38.2%, respectively, *P*<0.001).

The median age at the time of death was 11.1 months (interquartile range [IQR] 5.0, 22.7); 54% (n = 712) were male and 52% were infants (Table 1). Nearly two-thirds of deaths (62%, n = 819) occurred in healthcare facilities and 506 (38%) occurred in the community, outside the formal healthcare system. The most common causes of death determined by DeCoDe panels were lower respiratory tract infections (23%, n = 306), sepsis (20%, n = 262), and malnutrition (14%, n = 187) (Table A in S1 Text). The most common immediate cause of death was sepsis (15%, n = 194). The most common underlying cause of death was malnutrition (10%, n = 133) and the most common comorbid conditions were lower respiratory infections (9%, n = 114) and anemias (5%, n = 63).

**Table 1:** Demographics of infants and children who died in the Child Health and Mortality Prevention Surveillance network.

Characteristics	n (%)
**Age at Death in Months (median, IQR) (n = 1,321)** ^a^	11 (5, 23)
**Age Group at Death, (n = 1,326)**	
1 month to less <12 months old	685 (51.7)
12 months old to <60 months old	641 (48.3)
**Sex, (n = 1,324)** ^b^	
Male	712 (53.8)
Female	612 (46.2)
**Country of CHAMPS Site, (n = 1,326)**	
Bangladesh	78 (5.9)
Ethiopia	57 (4.3)
Kenya	287 (21.6)
Mali	235 (17.7)
Mozambique	256 (19.3)
Sierra Leone	257 (19.4)
South Africa	156 (11.8)
**Site of Death, (n = 1,325)** ^c^	
Healthcare Facility	819 (61.8)
Community	506 (38.2)
**Number of Causes of Death Determined by Minimally Invasive Tissue Sampling**^d^ **(n = 652)**	
Undetermined	10 (1.5)
1	140 (21.5)
2	207 (31.7)
3	154 (23.6)
≥4	141 (21.7)

^a^Five cases did not have an exact age at the time of death.

^b^Two cases did not have sex documented.

^c^One case was missing a location of death.

^d^Not all cases included underwent minimally invasive tissue sampling.

### Type of care sought

Nearly two-thirds of infants and children (64%, n = 851) had a hospital admission as their highest level of clinical care during the illness that led to their death, 214 (16%) had only one documented clinic visit prior to death, 39 (3%) only saw a traditional healer, and 133 (10%) received no formal clinical care in the immediate period prior to their death (Fig A in S1 Text). Among all infants and children, 31% (n = 412) sought care at both a clinic and a hospital, 7% (n = 95) sought care at a clinic, hospital, and traditional healer, and 22% (n = 290) sought care at only a hospital (Fig B in S1 Text). There were 16% (n = 208) of patients who only sought care at a clinic and 7% (n = 95) sought care at both a clinic and traditional healer.

The median time from symptom onset to seeking clinical care was 2 days (IQR 1, 4 days). Of 1,326 infants and children, 12% (n = 162) were found to be dead on arrival to a healthcare facility and 2% (n = 23) died during resuscitation shortly after arrival (Table 2). One-third (34%, n = 447) were seen at an outpatient clinic and not referred to a hospital; of those, 84% (n = 377) died at home without subsequent clinical encounters and 16% (n = 70) died in route to another healthcare facility. Approximately one-third (32%, n = 427) of infants and children were referred to a hospital after an outpatient encounter; of those, 10% (n = 41) died en route to the hospital and 19% (n = 82) died days later without reaching the referral facility. Among all 1,326 infant and child deaths evaluated, 739 (56%) died during hospitalization. There were 376 (28%) infants and children who were hospitalized, discharged, and died thereafter, with 86% (n = 325/376) of these dying in the community and 14% (n = 51/376) dying in another hospital after readmission.

**Table 2:** Clinical care received by deceased infants and children and their caregivers in the Child Health and Mortality Prevention Surveillance network (N = 1,326).

Characteristics	n (%)
**Received traditional healer care/medicines**	**282 (21.3)**
Any time in their clinical care course	243 (18.3)
Without receiving medical care	39 (2.9)
**Received over-the-counter medicine**	**232 (17.5)**
Any time in their clinical care course	190 (14.3)
Without receiving medical care	42 (3.2)
**Was dead on arrival to healthcare facility**	**162 (12.2)**
At an outpatient facility	62 (4.7)
At a hospital	100 (7.5)
**Died during resuscitation shortly after presentation**	**23 (1.7)**
Outpatient facility	2 (0.2)
Hospital	21 (1.6)
**Referred to hospital after outpatient visit**	**427 (32.2)**
Seen at any outpatient clinic, referred, but died en route	41 (3.1)
Seen at any outpatient clinic, referred, but did not go to referred facility and died	82 (6.2)
**Not referred to hospital after outpatient clinic**	**447 (33.7)**
Seen at any outpatient clinic, sent home, died en route to healthcare facility	70 (5.3)
Seen at outpatient and died at home without subsequent visits	377 (28.4)
**Children seen at a hospital, sent home, then died after**	**376 (28.4)**
Community	325 (86.4)
Another hospital	51 (13.6)
**Days to death after any clinical visit (median, IQR) (n = 649)**	**1 (0, 4)**
**Caregivers left clinical care against medical advice**	**15 (1.1)**
During outpatient visits	2 (0.2)
During hospital admission	13 (1.0)

### The Three Delays-in-Healthcare

**Fig 2:**
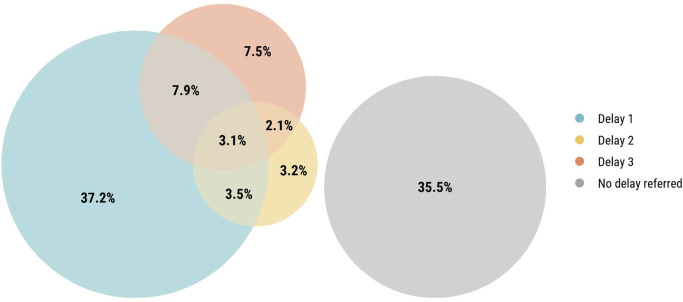
Venn diagram of each type of delay among deceased infants and children (N = 1,326).

**Fig 3:**
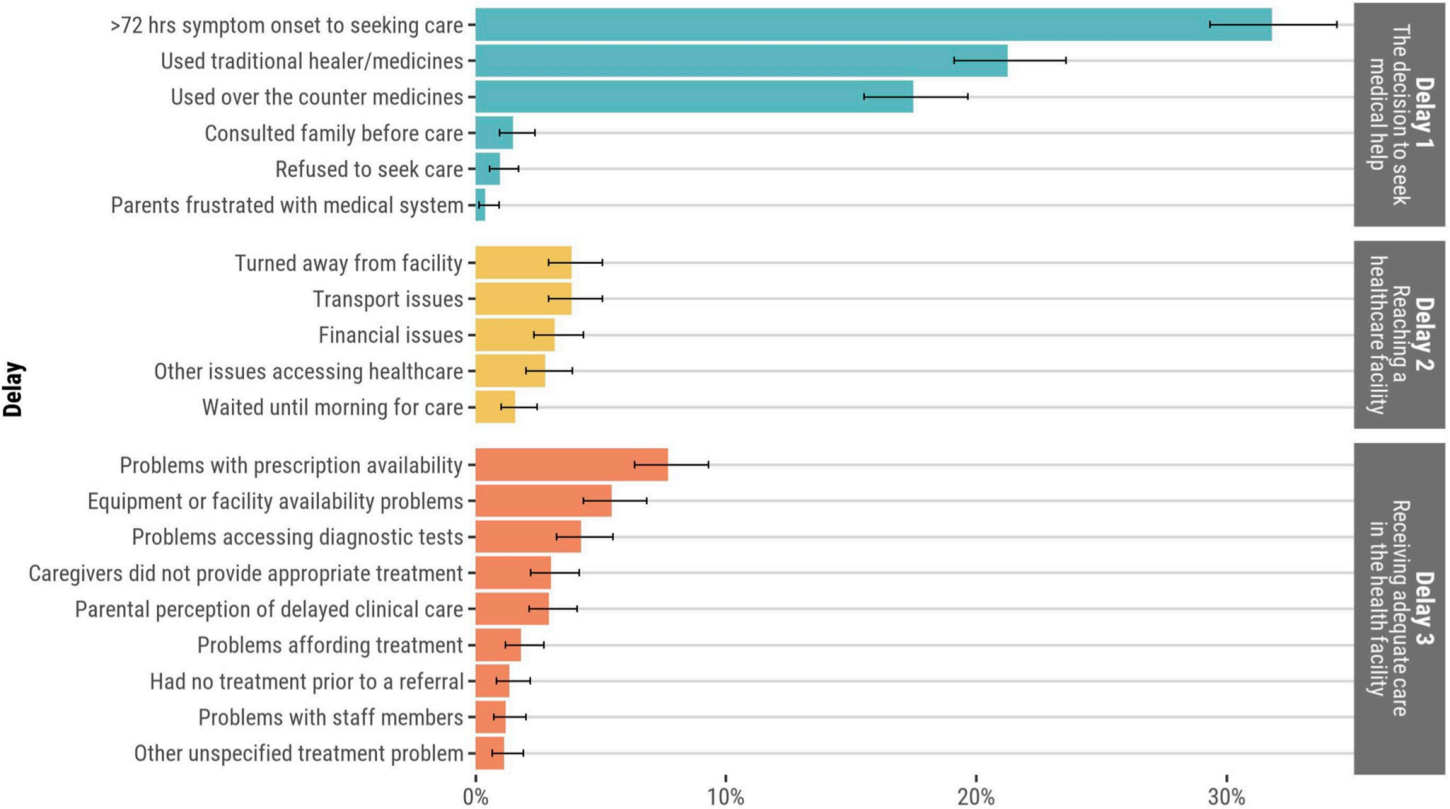
Proportion of deceased infants and children who experienced each delay in the “Three Delays-in-Healthcare” model.

Overall, 854/1,326 (64%) infants and children had at least one delay in the “Three Delays-in-Healthcare” framework that had been noted by families or in clinical records. However, only 3% (n = 41) had a delay in all the three categories (Fig 2). In the home, caregivers commonly waited >72 hours from symptom onset to deciding to seek clinical care (32%, n = 422). Also in the home, the preferred initial use of traditional medicines as therapy (21%, n = 282) and the use of over-the-counter medications (18%, n = 232) were common (Fig 3). Delays in attempting to reach a healthcare facility were less common overall. There were 4% (n = 51) who were turned away from a healthcare facility and 4% (n = 51) experienced challenges with transportation to healthcare facilities. In healthcare facilities, prescription medication unavailability (8%, n = 102), equipment unavailability or lack of bed availability to escalate care (5%, n = 72), and challenges accessing diagnostic tests (4%, n = 56) were most common.

**Fig 4:**
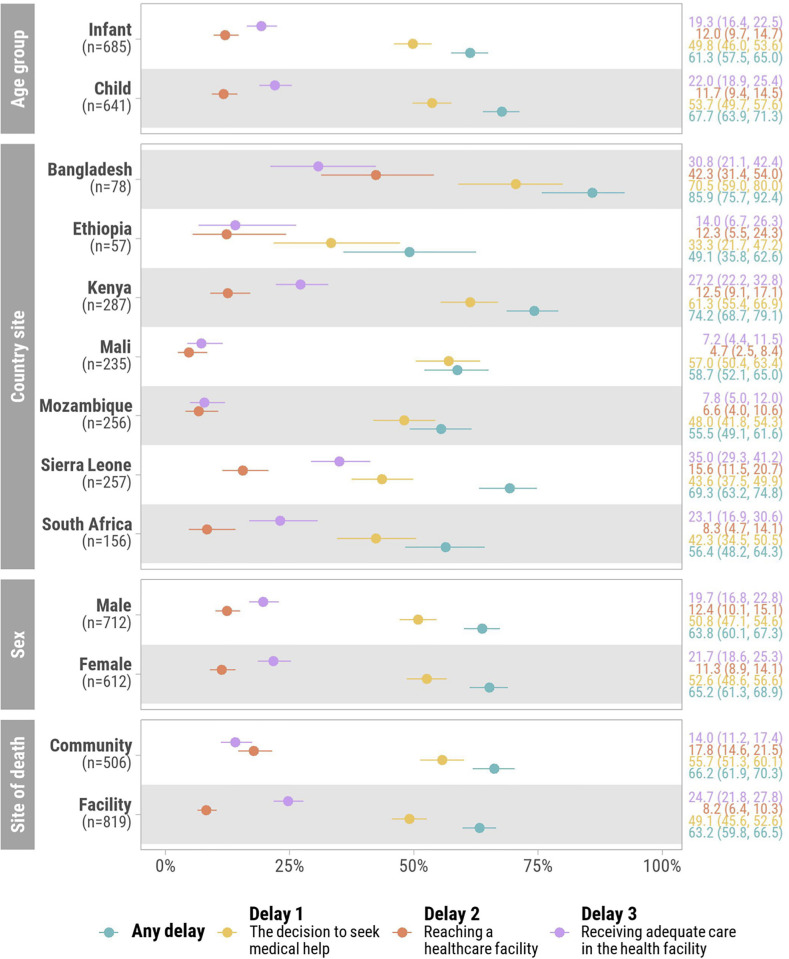
Proportion of infants and children who experienced each delay in “Three Delays-in-Healthcare” framework by age group, country site, sex, and site of death.

A slightly greater proportion of children aged 12–59 months experienced at least one delay compared to infants aged 1–11 months (68% vs. 61%, *P* = 0.018) (Fig 4). There were no significant differences in delays by participant sex. A minimally greater proportion of infants and children who died outside healthcare facilities experienced a delay in the decision to seek clinical care compared to those who died in a healthcare facility (74% vs 68%, *P* = 0.026).

**Fig 5:**
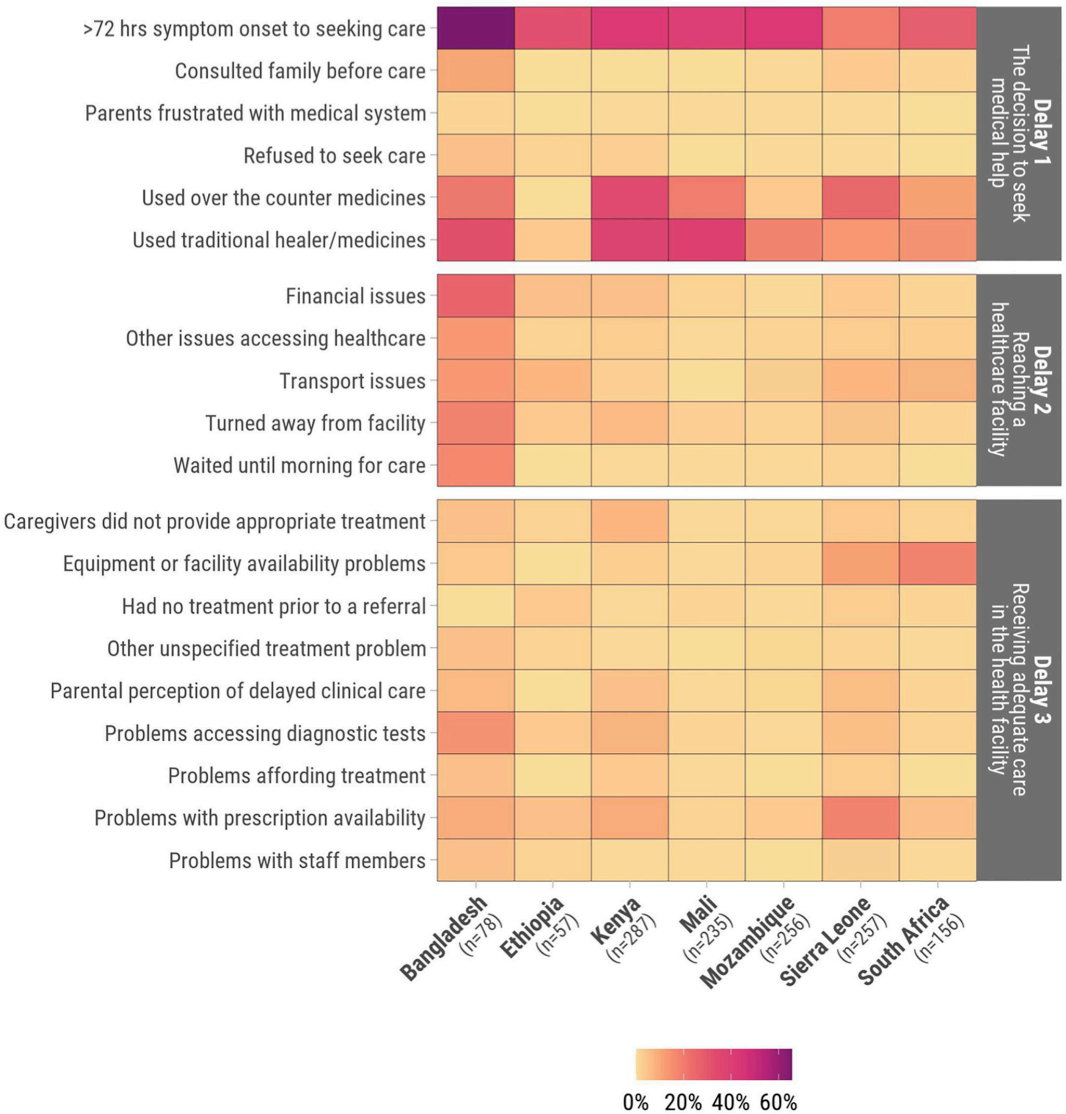
Proportion of delays in the “Three Delays-in-Healthcare” model among deceased infants and children by country site.

The proportion of infants and children who experienced any delay in the “Three Delays-in-Healthcare” model was highest in Bangladesh (86% n = 67/78) and Kenya (74%, n = 213/287), and lowest in Ethiopia (49%, n = 28/57) and Mozambique (56%, n = 142/256), with some variation seen in the type of delays encountered across sites (Table B in S1 Text). Waiting >72 hours to seek care was more common in Bangladesh (65%, n = 51/78) (*P*<0.001) than other sites (Fig 5). The preferred initial use of traditional medicine was most common in Mali (39.1%, n = 92/235), Kenya (37%, n = 107/287), and Bangladesh (32%, n = 25/78). Financial challenges for transport and paying for clinical care were common in Bangladesh (26%, n = 20/78). Deceased infants and children in Sierra Leone commonly faced challenges with prescription medication availability (18%, n = 46/257). Delays did not vary substantially by age in site-specific analyses (Fig C in S1 Text).

Among the 755 included cases that underwent full postmortem examinations to determine the cause of death, those who had a chronic condition (e.g., HIV, congenital birth defects, etc.) (69.3%, 232/335) were more likely to experience a delay in the home than those without a chronic condition (59.5%, 250/420; *P* = 0.007). There were no differences in delays in transport or receipt of clinical care between infants and children who had a chronic condition and those who did not. Children who died from HIV (n = 82, 83%) were more likely to experience at least one delay than those who died from meningitis/encephalitis (n = 41, 56%; *P* = 0.002; *q* = 0.048), congenital birth defects (n = 51, 57%; *P =* 0.001; *q* = 0.048), anemias (n = 95, 65%; *P =* 0.010; *q* = 0.092), lower respiratory infections (n = 306, 67%; *P =* 0.006; *q* = 0.086), and sepsis (n = 261, 67%; *P =* 0.008; *q* = 0.086) (Fig D in S1 Text). Children who had anemias as a cause of death (n = 95, 36%) encountered more delays in the provision of care compared to children with other diagnoses (*P* = 0.033).

## Discussion

Delays in healthcare seeking and provision were common among infants and children who died in seven sites in sub-Saharan Africa and Bangladesh, highlighting one aspect of the preventability of these deaths. Nearly two-thirds of deceased infants and children had at least one delay prior to their death. Delays in the home were more common than delays receiving clinical care. Deceased children aged 12–59 months experienced more delays than deceased infants aged 1–11 months. The frequency and type of delays varied by country where the infant or child died.

Similar to prior studies that evaluated healthcare seeking patterns for febrile infants and young children with pneumonia, caregivers frequently delayed seeking clinical care for >72 hours in our multi-site study [9, 22]. Our study extends these findings to several other conditions including sepsis, diarrheal diseases, malnutrition, and others. Thus, educational interventions or the use of health applications for caregivers on general warning signs and symptoms in which waiting >72 hours to seek care, or using over-the-counter medications alone, may be dangerous, and not just disease specific signs and symptoms, may be warranted [5].

Although delays in attempting to reach a healthcare facility were least common of the Three Delays in our study, transportation challenges were the most common type of delay among those under the category of delays “attempting to reach a healthcare facility”. This is consistent with prior studies demonstrating that transportation barriers may be common among both adult patients and caregivers of pediatric patients [9, 23, 32]. Thus, as health systems are strengthened in low- and middle-income countries, a focus on timely and cost-effective transportation to healthcare facilities, the provision of health care in transit (e.g., ambulances), and community-based health volunteers is needed. This may include expanded access to ambulances and other forms of transportation as has been done in India with the Referral Transportation System, which created a partnership between local governments and private vehicles by creating a centralized call center that connects patients with previously contracted private vehicles and provides free transportation services for patients in need [33]. Efforts are underway in the CHAMPS network in Mozambique to facilitate transportation to healthcare facilities. The impact of such efforts on childhood mortality should be measured in future work.

One-third of deceased patients were seen at an outpatient clinic and not referred to a hospital. There is previous evidence that in low-income countries, adequate triage and management of sick children remains insufficient and diagnostic uncertainty is frequent, contributing to raise the mortality burden [34–36]. In healthcare facilities, similar to our findings, prescription medication unavailability has been identified in prior studies in Malawi and India [30, 37]. Caregivers may not be aware of medication shortages in hospitals or clinics [22, 38], which could have led to an underestimate of medication shortages in our study given our data sources. Additionally, our finding that lack of bed availability for escalation of care and equipment availability issues were common is consistent with a study by Kakembo et al. in Uganda in which 56% of surgical patients had delayed surgery due to lack of hospital ward capacity and equipment issues [8]. Thus, structural support in healthcare systems including, but not limited to, transportation, road conditions, availability of skilled technicians, access to electricity may be needed to ensure adequate pharmacologic resources as well as equipment resources to improve clinical care in settings similar to those in the CHAMPS network [39, 40].

Similar to prior studies [25, 26, 41], deceased children aged 12–59 months had more delays than infants aged 1–11 months in our study. This may suggest greater caregiver concern for younger infants who are unable to localize pain or describe symptoms. Alternatively, there could be greater concern for the fragility of infants compared to older children.

We found no significant differences in healthcare seeking delays between deaths occurring in male infants and children compared to females. Prior work evaluating healthcare seeking delays by sex has shown mixed results with some suggesting delays are more common for female children and others not demonstrating the same difference [24, 26–28, 42]. As these prior studies focused on non-fatal conditions, our findings may suggest that healthcare seeking behaviors are not different by sex for severe illness.

There was a significant difference in the number and type of delays among deceased infants and children by site. This was anticipated given the variability in resources and transportation options between sites. Few studies have been designed to allow cross-country comparisons of delays in healthcare seeking [8, 11, 23]. Caregivers at each site shared similar patterns in healthcare seeking delays in the home, which points toward widespread need for improved education on when to seek clinical care for infants and children. Culture, context, and feasibility need to be accounted for when developing such interventions. For example, in countries with higher initial preferred use of traditional medicine, such as Mali, Kenya, and Bangladesh, public health officials and clinicians may consider partnering with traditional healers to disseminate information on when to refer to a higher level of care [43–46]. However, direct comparisons between countries are difficult given variability in urbanicity, resources in healthcare facilities, and cost of clinical care.

### Limitations

We could only account for delays in healthcare seeking that were described in clinical summaries or shared by caregivers after their infant or child died, which may have been subject to recall bias, particularly when discussing a distressing topic such as the death of their infant or child. Delays not described in those narratives may still have existed (e.g., provision of counterfeit medications, out of pocket costs or indirect costs for clinical care, distance to healthcare facilities, or other structural factors that may impact delays in care seeking as described in previous studies) [47, 48]. Given the retrospective nature of this study, we did not have detailed information about distances from homes to healthcare facilities or the socioeconomic status of families of deceased infants and children, which precluded our ability to comment on the impact of distance as a barrier to seeking healthcare or the impact of socioeconomic barriers on healthcare seeking behaviors. We were also limited in the number of children included in each cause of death, which resulted in small numbers in the comparison of delays by diagnoses. There are no traditional controls in CHAMPS so there was no comparison to children who did not die, making it impossible to draw conclusions about delays definitively leading to mortality among infants and children. These data were collected prior to the availability of the 2022 WHO Verbal Autopsy form, which may reduce the duration of the interview through improved question wording and flow [49]. Additional studies may be warranted to assess delays in care seeking using the updated WHO Verbal Autopsy form. Lastly, as caregivers and clinicians were not asked specific details about each component of the “Three Delays-in-Healthcare” framework, our findings may underestimate the true frequency of these delays.

## Conclusions

Delays in healthcare seeking and provision were common among deceased infants and children in seven regions with high childhood mortality rates in sub-Saharan Africa and Bangladesh. Community-based interventions to prompt timely healthcare seeking should be studied as a potential approach to reduce mortality among infants and children. Efforts are needed to ensure timely, affordable, and safe transportation to healthcare facilities and to address stock-outs and equipment maintenance, so that indicated medications and functioning equipment are available in healthcare facilities to ensure high-quality clinical care for infants and children. It is imperative that solutions and interventions account for both regional and national needs given the differences in delays experienced by children depending on their country of origin.

## Supporting information

S1 Text **Tables A, B and Figs A-D. Table A**. Summary of DeCoDed causes of death for infants and children aged 1–59 months who died in the CHAMPS network (N = 1,326). **Table B**. Proportion of deaths that had experienced each delay in the 3 delays model by site, sex, age group, causes of death, and site of death, N = 1,326. **Fig A**. Highest level of healthcare facilities in which deceased infants and children received clinical (N = 1,326). **Fig B**. Venn diagram representing the intersectionality of care for infants and children in the CHAMPS Network between outpatient clinical visits, hospital clinical visits and traditional healing (N = 1,326). **Fig C**. Proportion of delays in the “Three Delays-in-Healthcare” model among deceased infants and children by country site by age. **Fig D**. Frequencies of delays in the 3 delays model (A) and frequencies of specific challenges (B) for the top ten DeCoDed causes of death anywhere in the causal chain.
(DOCX)

S2 Text STROBE checklist. (DOC)

S1 Acknowledgments CHAMPS consortium members. (DOCX)
