# Supplementary material for: Identifying delays in healthcare seeking and provision: The Three Delays-in-Healthcare and mortality among infants and children aged 1–59 months
Source: PLOS Glob Public Health. 2024 Feb 8;4(2):e0002494. doi: 10.1371/journal.pgph.0002494 (PMC10852234; doi:10.1371/journal.pgph.0002494)
Supplement: S1 Text — Tables A, B and Figs A-D. Table A. Summary of DeCoDed causes of death for infants and children aged 1–59 months who died in the CHAMPS network (N = 1,326). Table B. Proportion of deaths that had experienced each delay in the 3 delays model by site, sex, age group, causes of death, and site of death, N = 1,326. Fig A. Highest level of healthcare facilities in which deceased infants and children received clinical (N = 1,326). Fig B. Venn diagram representing the intersectionality of care for infants and children in the CHAMPS Network between outpatient clinical visits, hospital clinical visits and traditional healing (N = 1,326). Fig C. Proportion of delays in the “Three Delays-in-Healthcare” model among deceased infants and children by country site by age. Fig D. Frequencies of delays in the 3 delays model (A) and frequencies of specific challenges (B) for the top ten DeCoDed causes of death anywhere in the causal chain. (DOCX) [file pgph.0002494.s001.docx]

**Table A in S1 Text**. Summary of DeCoDe causes of death for infants and children aged 1-59 months who died in the CHAMPS network (N=1,326)

| **Cause of Death (DeCoDe), n (%)** | **Overall, n (%)** | **Immediate Cause of Death, n (%)** | **Underlying Cause of Death, n (%)** | **Comorbid Cause of Death, n (%)** |
| --- | --- | --- | --- | --- |
| Anemias | 95 (7.2) | 31 (2.3) | 1 (0.1) | 63 (4.8) |
| Cancer | 4 (0.3) | 0 (0) | 4 (0.3) | 0 (0) |
| Congenital birth defects | 51 (3.8) | 0 (0) | 47 (3.5) | 9 (0.7) |
| Congenital infection | 2 (0.2) | 0 (0) | 2 (0.2) | 0 (0) |
| Diabetes | 1 (0.1) | 1 (0.1) | 1 (0.1) | 0 (0) |
| Diarrheal Diseases | 109 (8.2) | 35 (2.6) | 54 (4.1) | 31 (2.3) |
| Epilepsy | 3 (0.2) | 1 (0.1) | 2 (0.2) | 0 (0) |
| Heart Diseases | 5 (0.4) | 2 (0.2) | 1 (0.1) | 2 (0.2) |
| HIV | 82 (6.2) | 2 (0.2) | 82 (6.2) | 2 (0.2) |
| Injury | 28 (2.1) | 2 (0.2) | 24 (1.8) | 2 (0.2) |
| Kidney Disease | 3 (0.2) | 0 (0) | 0 (0) | 3 (0.2) |
| Liver disease | 9 (0.7) | 4 (0.3) | 4 (0.3) | 4 (0.3) |
| Lower respiratory infections | 306 (23.1) | 140 (10.6) | 67 (5.1) | 114 (8.6) |
| Malaria | 163 (12.3) | 54 (4.1) | 95 (7.2) | 36 (2.7) |
| Malnutrition | 187 (14.1) | 2 (0.2) | 133 (10.0) | 52 (3.9) |
| Measles | 2 (0.2) | 0 (0) | 2 (0.2) | 0 (0) |
| Meningitis/Encephalitis | 41 (3.1) | 13 (1.0) | 3 (0.2) | 26 (2.0) |
| Motor neuron disease | 1 (0.1) | 0 (0) | 1 (0.1) | 0 (0) |
| Neonatal preterm birth complications | 16 (1.2) | 0 (0) | 15 (1.1) | 3 (0.2) |
| Other | 27 (2.0) | 3 (0.2) | 5 (0.4) | 19 (1.4) |
| Other disorders of fluid, electrolyte and acid-base balance | 5 (0.4) | 2 (0.2) | 0 (0) | 3 (0.2) |
| Other endocrine, metabolic, blood, and immune disorders | 13 (1.0) | 2 (0.2) | 5 (0.4) | 6 (0.5) |
| Other gastrointestinal disease | 1 (0.1) | 0 (0) | 0 (0) | 1 (0.1) |
| Other immunodeficiencies | 4 (0.3) | 0 (0) | 1 (0.1) | 3 (0.2) |
| Other infections | 37 (2.8) | 11 (0.8) | 12 (0.9) | 18 (1.4) |
| Other neonatal disorders | 6 (0.5) | 0 (0) | 4 (0.3) | 2 (0.2) |
| Other neurological disorders | 18 (1.4) | 1 (0.1) | 7 (0.5) | 11 (0.8) |
| Other respiratory disease | 59 (4.4) | 32 (2.4) | 10 (0.8) | 21 (1.6) |
| Other skin and subcutaneous diseases | 6 (0.5) | 0 (0) | 3 (0.2) | 3 (0.2) |
| Paralytic ileus and intestinal obstruction | 4 (0.3) | 0 (0) | 2 (0.2) | 3 (0.2) |
| Poisoning | 7 (0.5) | 4 (0.3) | 2 (0.2) | 2 (0.2) |
| Rabies | 1 (0.1) | 0 (0) | 1 (0.1) | 0 (0) |
| Sepsis | 262 (19.8) | 194 (14.6) | 38 (2.9) | 42 (3.2) |
| Sickle cell disorders | 7 (0.5) | 0 (0) | 5 (0.4) | 2 (0.2) |
| Sudden infant death syndrome | 1 (0.1) | 0 (0) | 1 (0.1) | 0 (0) |
| Syphilis | 1 (0.1) | 1 (0.1) | 1 (0.1) | 0 (0) |
| Tuberculosis | 7 (0.5) | 3 (0.2) | 4 (0.3) | 2 (0.2) |
| Undetermined | 10 (0.8) | 0 (0) | 10 (0.8) | 0 (0) |
| Upper respiratory infections | 4 (0.3) | 1 (0.1) | 2 (0.2) | 2 (0.2) |

| **Table B in S1 Text**. Proportion of deaths that had experienced each delay in the 3 delays model by site, sex, age group, causes of death, and site of death, N=1,326 | | | | | | | | | |
| --- | --- | --- | --- | --- | --- | --- | --- | --- | --- |
|  | **Deaths** | **Delay 1** | | **Delay 2** | | **Delay 3** | | **Any delay** | |
|  | **N** | **N (%)** | **P-value*** | **N (%)** | **P-value** | **N (%)** | **P-value** | **N (%)** | **P-value** |
| **Site** |  |  | **<0.001** |  | **<0.001** |  | **<0.001** |  | **<0.001** |
| Bangladesh | 78 | 55 (70.5) |  | 33 (42.3) |  | 24 (30.8) |  | 67 (85.9) |  |
| Ethiopia | 57 | 19 (33.3) |  | 7 (12.3) |  | 8 (14.0) |  | 28 (49.1) |  |
| Kenya | 287 | 176 (61.3) |  | 36 (12.5) |  | 78 (27.2) |  | 213 (74.2) |  |
| Mali | 235 | 134 (57.0) |  | 11 (4.7) |  | 17 (7.2) |  | 138 (58.7) |  |
| Mozambique | 256 | 123 (48.0) |  | 17 (6.6) |  | 20 (7.8) |  | 142 (55.5) |  |
| Sierra Leone | 257 | 112 (43.6) |  | 40 (15.6) |  | 90 (35.0) |  | 178 (69.3) |  |
| South Africa | 156 | 66 (42.3) |  | 13 (8.3) |  | 36 (23.1) |  | 88 (56.4) |  |
| Sex (N=1,324) |  |  | 0.557 |  | 0.601 |  | 0.390 |  | 0.628 |
| Male | 712 | 362 (50.8) |  | 88 (12.4) |  | 140 (19.7) |  | 454 (63.8) |  |
| Female | 612 | 322 (52.6) |  | 69 (11.3) |  | 133 (21.7) |  | 399 (65.2) |  |
| Age group |  |  | 0.174 |  | 0.946 |  | 0.246 |  | 0.018 |
| Infant | 685 | 341 (49.8) |  | 82 (12.0) |  | 132 (19.3) |  | 420 (61.3) |  |
| Child | 641 | 344 (53.7) |  | 75 (11.7) |  | 141 (22.0) |  | 434 (67.7) |  |
| Site of death (N=1,325) |  |  | 0.029 |  | <0.001 |  | <0.001 |  | 0.373 |
| Community | 506 | 282 (55.7) |  | 90 (17.8) |  | 71 (14.0) |  | 335 (66.2) |  |
| Facility | 819 | 402 (49.1) |  | 67 (8.2) |  | 202 (24.7) |  | 518 (63.2) |  |

*****Comparisons made among each subgroup using chi-square test

**Fig A in S1 Text**. Highest level of healthcare facilities in which deceased infants and children received clinical (N=1,326)

**
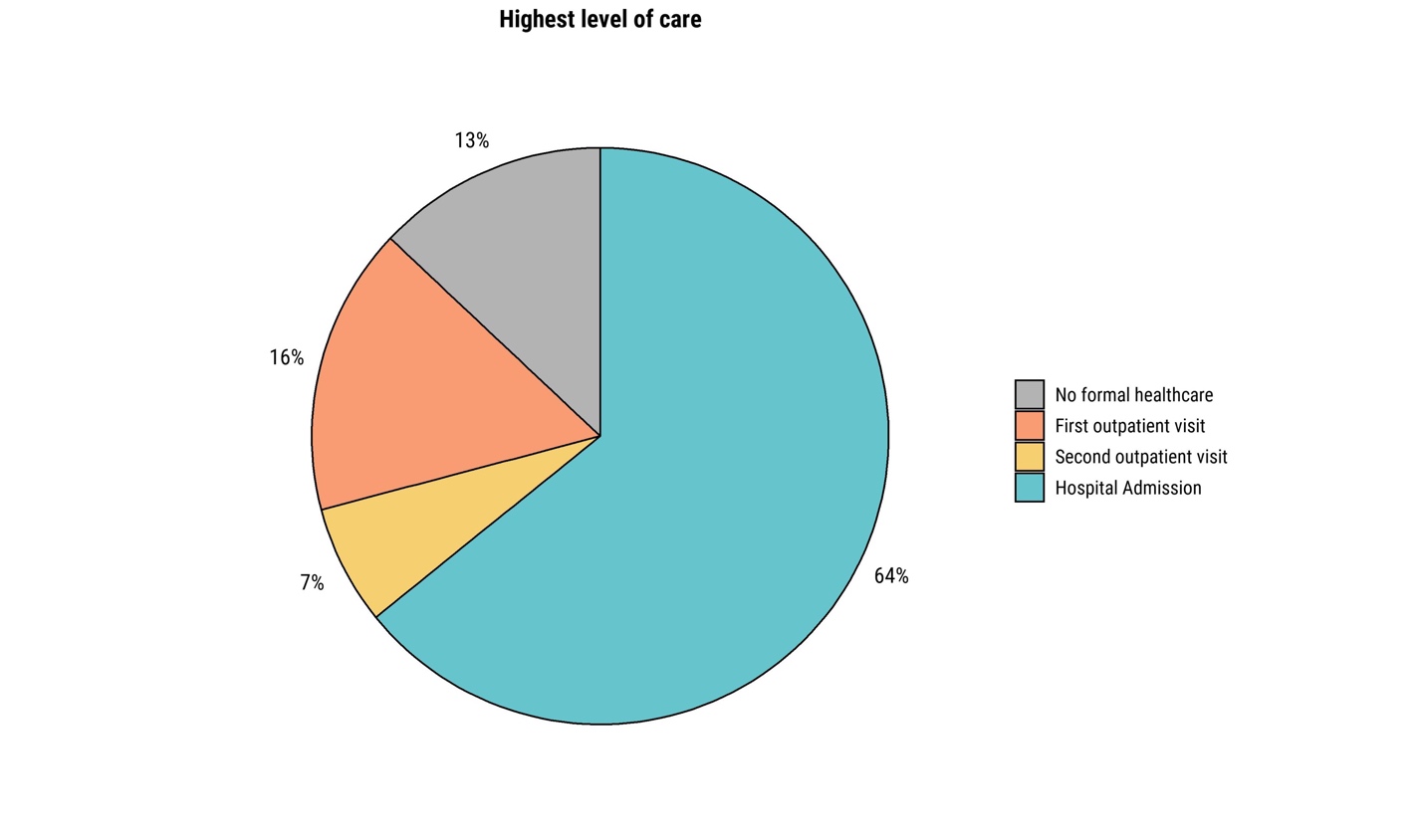
Fig B in S1 Text.** Venn diagram representing the intersectionality of care for infants and children in the CHAMPS Network between outpatient clinical visits, hospital clinical visits and traditional healing (N=1,326)


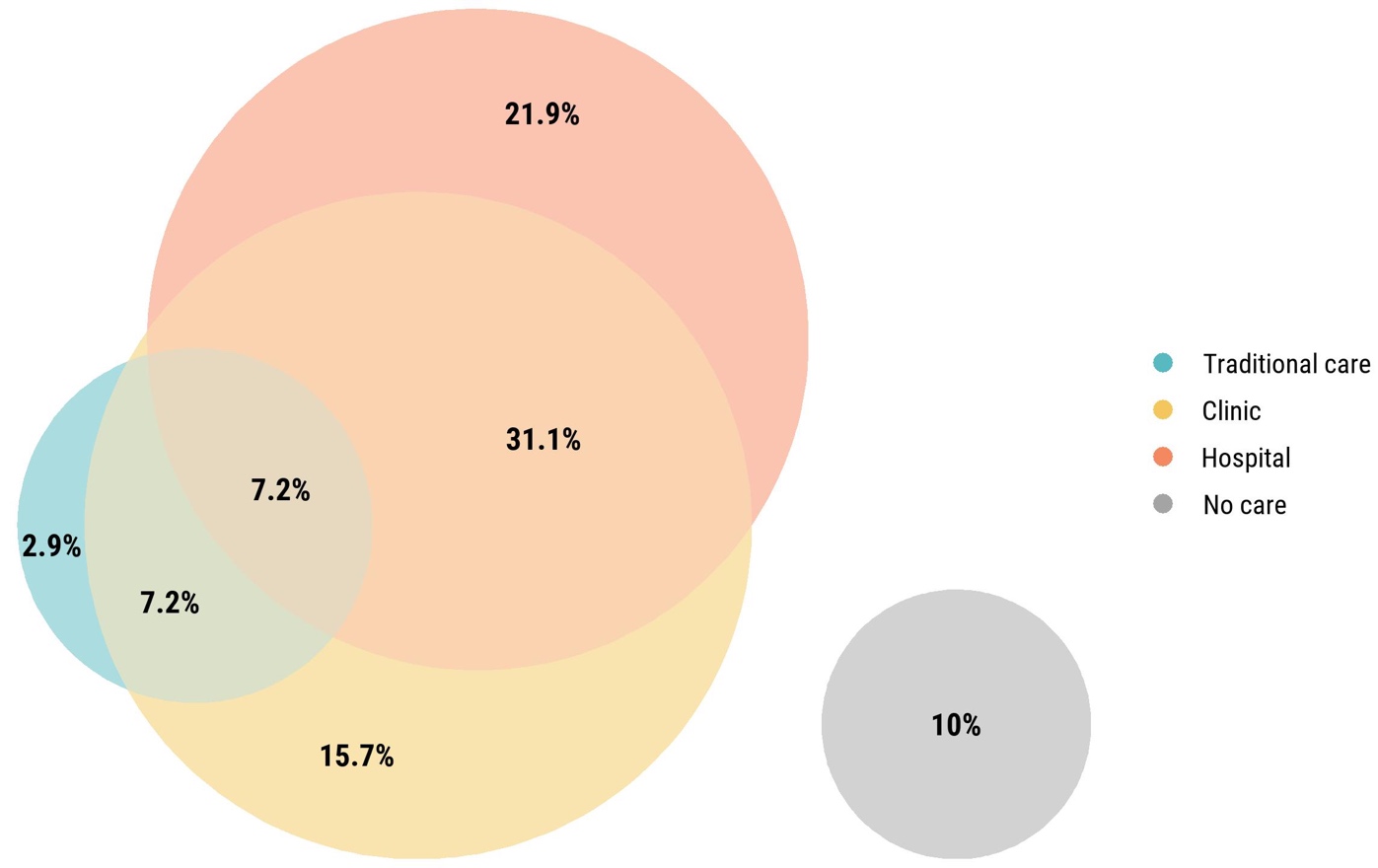


**Fig C in S1 Text.** Proportion of delays in the “Three Delays-in-Healthcare” model among deceased infants and children by country site by age

**
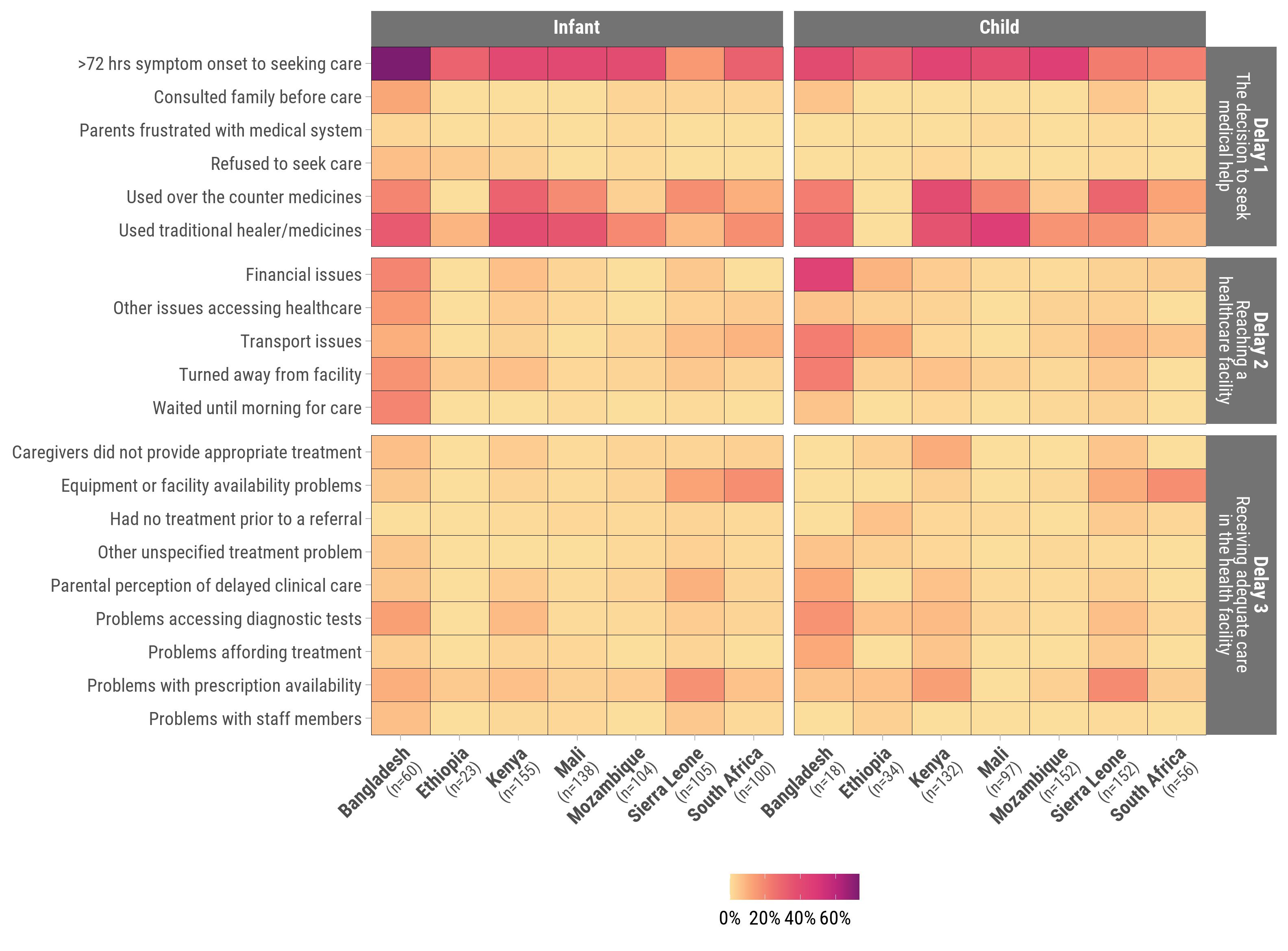
**

**Fig D in S1 Text.** Frequencies of delays in the 3 delays model (A) and frequencies of specific challenges (B) for the top ten DeCoDed causes of death anywhere in the causal chain

**
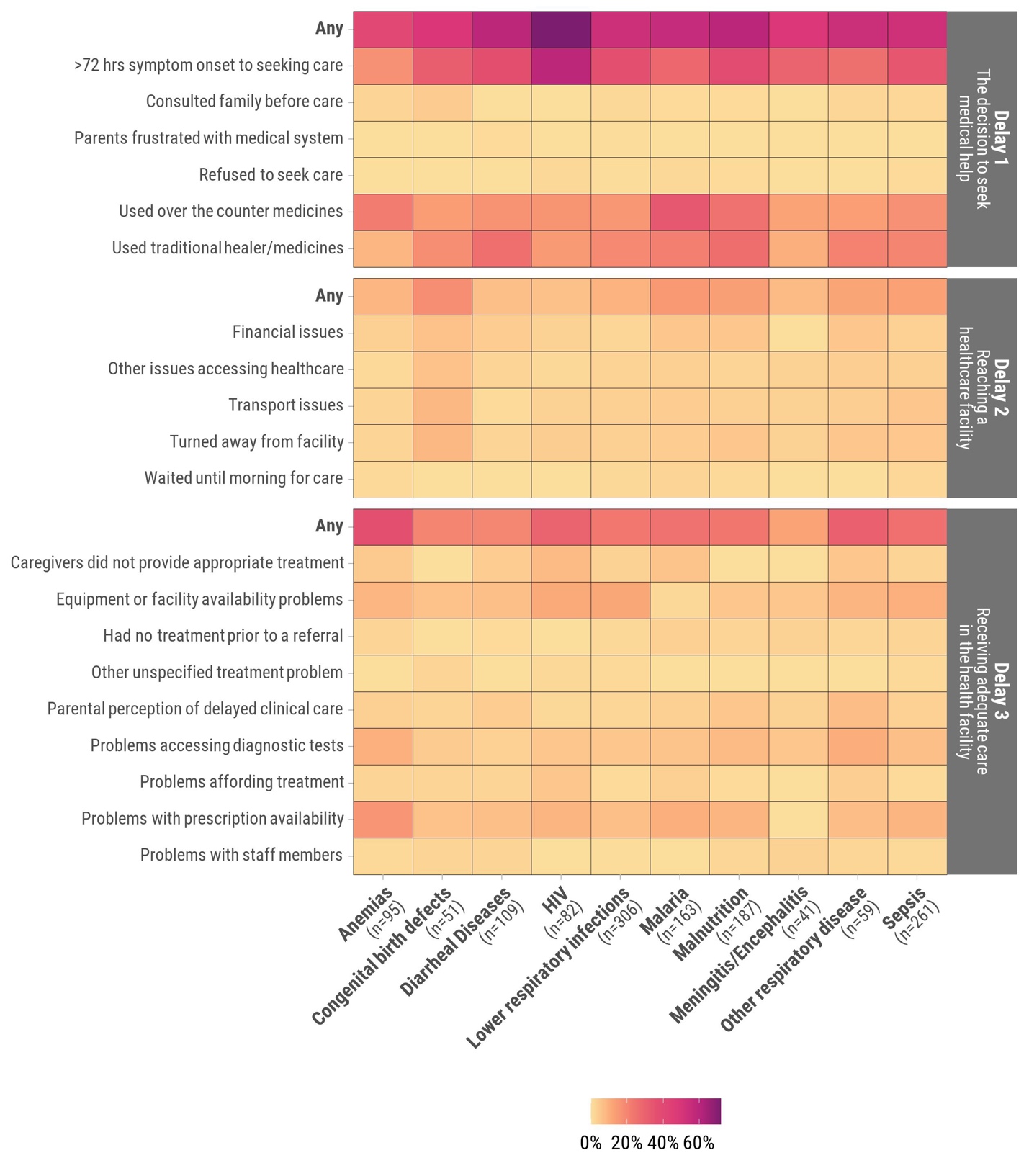
**
