# Supplementary material for: Identifying delays in healthcare seeking and provision: The Three Delays-in-Healthcare and mortality among infants and children aged 1–59 months
Source: PLOS Glob Public Health. 2024 Feb 8;4(2):e0002494. doi: 10.1371/journal.pgph.0002494 (PMC10852234; doi:10.1371/journal.pgph.0002494)
Supplement: S1 Acknowledgments — (DOCX) [file pgph.0002494.s003.docx]

**S1 Acknowledgements**. CHAMPS Consortium Members

| **First names** | **Surnames** |
| --- | --- |
| Fatima | Solomon |
| Gillian | Sorour |
| Hennie | Lombaard |
| Jeannette | Wadula |
| Karen | Petersen |
| Martin | Hale |
| Nelesh P. | Govender |
| Peter J. | Swart |
| Sanjay G. | Lala |
| Sithembiso | Velaphi |
| Richard | Chawana |
| Yasmin | Adam |
| Ashleigh | Fritz |
| Nellie | Myburgh |
| Pedzisai | Ndagurwa |
| Cleopas | Hwinya |
| Sanwarul | Bari |
| Shahana | Parveen |
| Mohammed | Kamal |
| A.S.M. Nawshad | Uddin Ahmed |
| Mahbubul | Hoque |
| Saria | Tasnim |
| Ferdousi | Islam |
| Farida | Ariuman |
| Mohammad Mosiur | Rahman |
| Ferdousi | Begum |
| K. | Zaman |
| Mustafizur | Rahman |
| Dilruba | Ahmed |
| Meerjady Sabrina | Flora |
| Tahmina | Shirin |
| Mahbubur | Rahman |
| Joseph | Oundo |
| Alexander M. | Ibrahim |
| Fikremelekot | Temesgen |
| Tadesse | Gure |
| Addisu | Alemu |
| Melisachew Mulatu | Yeshi |
| Mahlet Abayneh | Gizaw |
| Stian MS | Orlien |
| Solomon | Ali |
| Peter | Otieno |
| Peter Nyamthimba | Onyango |
| Janet | Agaya |
| Richard | Oliech |
| Joyce Akinyi | Were |
| Dickson | Gethi |
| Sammy | Khagayi |
| George | Aol |
| Thomas | Misore |
| Harun | Owuor |
| Christopher | Mugah |
| Bernard | Oluoch |
| Christine | Ochola |
| Sharon M. | Tennant |
| Carol L. | Greene |
| J. Kristie | Johnson |
| Brigitte | Gaume |
| Rima | Koka |
| Karen D. | Fairchild |
| Diakaridia | Kone |
| Sharon M. | Tennant |
| Ashka | Mehta |
| Doh | Sanogo |
| Uma U. | Onwuchekwa |
| Nana | Kourouma |
| Seydou | Sissoko |
| Cheick Bougadari | Traore |
| Jane | Juma |
| Kounandji | Diarra |
| Awa | Traore |
| Tiéman | Diarra |
| Kiranpreet | Chawla |
| Tacilta | Nhampossa |
| Zara | Manhique |
| Sibone | Mocumbi |
| Clara | Menéndez |
| Khátia | Munguambe |
| Maria | Maixenchs |
| Andrew | Moseray |
| Fatmata Bintu | Tarawally |
| Martin | Seppeh |
| Ronald | Mash |
| Julius | Ojulong |
| Babatunde | Duduyemi |
| James | Bunn |
| Alim | Swaray-Deen |
| Joseph | Bangura |
| Amara | Jambai |
| Margaret | Mannah |
| Okokon | Ita |
| Cornell | Chukwuegbo |
| Sulaiman | Sannoh |
| Princewill | Nwajiobi |
| Dickens | Kowuor |
| Erick | Kaluma |
| Oluseyi | Balogun |
| Solomon | Samura |
| Samuel | Pratt |
| Francis | Moses |
| Tom | Sesay |
| James | Squire |
| Joseph Kamanda | Sesay |
| Osman | Kaykay |
| Binyam | Halu |
| Francis | Smart |
| Sartie | Kenneh |
| Soter | Ameh |
| Sartie | Kenneh |
| Jana | Ritter |
| Tais | Wilson |
| Jonas | Winchell |
| Jakob | Witherbee |
| Navit T. | Salzberg |
| Jeffrey P. | Koplan |
| Margaret | Basket |
| Ashutosh | Wadhwa |
| Kyu Han | Lee |
| Valentine | Wanga |
| Roosecelis | Martines |
| Shamta | Warang |
| Maureen | Diaz |
| Jessica | Waller |
| Shailesh | Nair |
| Lucy | Liu |
| Courtney | Bursuc |
| Kristin | LaHatte |
| Sarah | Raymer |
| John | Blevins |
| Solveig | Argeseanu |
| Kurt | Vyas |
| Manu | Bhandari |
